# Corneal transplant during COVID-19 pandemic: the Italian Eye Bank national report

**DOI:** 10.1007/s10561-021-09934-8

**Published:** 2021-05-24

**Authors:** Francesco Aiello, Federico Genzano Besso, Giulio Pocobelli, Gabriele Gallo Afflitto, Rossella Anna Maria Colabelli Gisoldi, Carlo Nucci, Diego Ponzin, on behalf of Italian Society Eye Bank Group (SIBO)., Paola Bonci, Giuseppe Calabrò, Roberto Ceccuzzi, Massimiliano Corneli, Germano Genitti, Claudio Giannarini, Raffaela Mistò, Augusto Pocobelli, Achille Tortori, Davide Venzano

**Affiliations:** 1 Ophthalmology Unit. Department of Experimental Medicine, University of Rome “Tor Vergata”, Rome, Italy; 2 Eye Bank of Piedmont, SSD Tissue Banks and Biorepository, Città della Salute e della Scienza, Torino, Italy; 3 Ophthalmology Unit, San Giovanni Addolorata Hospital, Rome, Italy; 4 The Veneto Eye Bank Foundation, Venice, Italy

**Keywords:** Eye banks activities, Corneal transplant, COVID-19, SARS-CoV-2, Lockdown

## Abstract

To investigate the impact of Coronavirus Disease-2019 lockdown on the Italian Eye Bank organization. In this national retrospective, multicentric, cohort study, data from the Italian Eye Bank during both the lockdown and the first month after the lockdown period were retrieved. We compared the Italian Eye Bank metrics with the same timeframe of 2019 and 2018. Data from 13 out of 13 (100%) Italian Eye Banks were included in the analysis. A statistically significant reduction in the number of donor corneas retrieved in 2020 was found as compared to the same period in 2019 and in 2018, respectively (2020 = 1284; 2019 = 3088; 2018 = 3221; ANOVA: *p* < 0.0001). Only 534 corneas have been distributed by Eye Banks during the COVID-19-lockdown period (2020 = 534; 2019 = 1220; 2018 = 1237. ANOVA: *p* < 0.0001). Similarly, the number of wasted corneas due to postponed or cancelled surgeries was 421, resulting in a considerable increase as compared to the previous 2 years (2020 = 421; 2019 = 67; 2018 = 84; ANOVA: *p* = 0.0035). Overall, 45 donor corneas were rejected in accordance with the guidance of the Italian National Health Institute Italian National Transplant Centre (CNT). SARS-CoV-2 pandemic has profoundly affected every social and medical field, including the Eye Bank procurement and distribution programs. The current data collected from all the Italian Eye Banks highlights the present and the forthcoming difficulties that the Eye Bank community is going to experience, as for the ongoing pandemic.

## Introduction

On December 31, 2019, China notified the World Health Organization (WHO) of a pneumonia outbreak of unknown aetiology.The causative agent was named as Severe Acute Respiratory Syndrome-Coronavirus-2 (SARS-CoV-2), responsible for the Coronavirus Disease-2019 (COVID-19). (Zhu et al. 2020; Aiello et al. 2020).

Italy was the first western European country affected by the pandemic. As of March 9, 2020, the Italian Government imposed a national quarantine, restricting the movements of the population in response to the growing epidemic of COVID-19. (Buonomo et al. 2020) This decision drastically reduced healthcare provision, with disruption and cancellation of non-urgent medical procedures, including elective corneal transplant surgeries (Olivia Li et al. 2020; Vanni et al. 2020b; Busin et al. 2020) Gain et al. demonstrated that the Italian Eye Bank Institution is the second main procurer of corneal grafts after the United States of America, with 250•10^–6^ cornea donations per capita per year, rendering it one of the most prolific Eye Bank Institution throughout the world. (Gain et al. 2016).

Hence, the aim of this study is to analytically explore the COVID-19 impact on the Italian Eye Bank organization, as a reliable index of the COVID-19 effect on the Eye Bank activity worldwide.

## Material and methods

We collected data from the Italian Society of Eye Banks (SIBO), the reference board in charge of the coordination of the 13 Eye Banks in Italy (Società Italiana Banche degli Occhi 2000).

We compared the number of corneal transplant procedures performed during the Italian lockdown period (March to April 2020) together with the first month post lockdown (May 2020) as compared with the same timeframe in both 2019 and 2018. All the available information regarding the number, type and setting (i.e., emergency *vs.* elective procedure) of performed corneal transplant surgeries was analysed. In addition, we investigated the number of wasted tissues (recovered, processed and expired) and the number of tissues collected from donors who were subsequently found to be positive to COVID-19. Furthermore, we collected data regarding the number of corneas already stored in the eye banks that had expired and were wasted, as well as the number of tissues that have been booked by surgeons but sent back due to unexpected cancellation in 2020, 2019 and in 2018 during the same aforementioned months.

We also evaluated the eye bank activity in the month following the end of the lockdown period compared with the one of both 2019 and 2018, as an index of the resumption of both clinical and surgical activity throughout the country. Finally, we compared the corneal transplant surgical activity during the lockdown period with the one performed in the subsequent month. Each eye bank provided anonymised and aggregate data and thus the institutional review board ruled that ethics approval was not required for this study.

## Statistical analysis

The statistical analysis was performed using the SPSS software version 26.0 (SPSS Inc.). Unmatched, non-parametric continuous variables were compared using the Mann–Whitney test. The Wilcoxon matched-pairs signed rank test was used to compare matched, non-parametric continuous variables. Analysis of Variance (ANOVA) test was used for unmatched, parametric, continuous variables. Friedman test was used for matched, non-parametric, continuous variables. For unadjusted comparisons, a 2-sided α of less than 0.05 was considered statistically significant.

## Results

In this national multicentre, retrospective cohort study, data from 13 out of 13 (100%) Italian eye banks were included in the analysis. Notably, Eye Banks from northern Italy (Lombardia, Veneto, Piemonte and Emilia-Romagna), located in those Italian regions more affected by COVID-19 pandemic, were adequately represented, accounting for the 38% of the entire cohort. Eye Banks from central and southern Italy accounted for the 46% and the 15%, respectively.

## Italian corneal transplant machinery data during the lockdown period

Overall, from the 1st of March, 2020 to the 30th of April, 2020, (months of lockdown) 1284 donor corneas had been collected and harvested. After proper investigation, only 31 grafts (2%) were found to not be feasible to transplant due to the risk of Sars-CoV-2 contamination, in accordance to the guidance of the Italian National Health Institute/Italian National Transplant Centre (CNT) (donor with proved COVID-19 or donor with unproved infection but pathological history consistent of COVID-19). (Istituto Superiore di Sanità 2020) A statistically significant reduction was found in the number of retrieved corneas in 2020 as compared with the one relative to the same period in 2019 (− 58%) and in 2018 (− 60%), respectively (Table 1).

**Table 1:** Corneal transplant metrics during the lockdown period (March and April, 2020) as compared to the same timeframe in 2019 and 2018

	March–April 2020	March–April 2019	March–April 2018	*p*
N° of received corneas	1284	3088	3221	< 0.0001
N° of Surgical Procedures	534	1220	1237	< 0.0001
N° of PKs	247	519	582	< 0.0001
N° of ALKs	49	188	158	0.0013
N° of EKs	238	571	572	< 0.0001
N° of EPs	30	59	46	0.1282
N° of cancelled procedures	103	27	35	0.0001
N° of requested albeit not transplanted corneas	9	26	24	0.1024
N° of waisted grafts	421	67	84	0.0035

*PK* penetrating keratoplasty; *ALK* Anterior lamellar keratoplasty; *EK* endothelial keratoplasty

In Italy, only 534 corneas were distributed during the COVID-19-lockdown period, with a significant reduction when compared to those of 2019 (− 56%) and 2018 (− 57%). In particular, 247 (46%) penetrating keratoplasties (PKs), 49 (10%) anterior lamellar keratoplasties (ALKs) and 238 (45%) endothelial keratoplasties (EKs) were recorded. Significant differences emerged from the comparison of 2020 PKs, ALKs and EKs data with both the 2019 and the 2018 ones, as shown in Table 1 (PK reduction: − 52% in 2019 and − 58% in 2018; ALK reduction: − 74% in 2019 and − 69% in 2018; EK reduction: − 58 in 2019 and − 58% in 2018).

Thirty (5%) of those 534 surgeries were performed as emergency procedures (EP) with no significant difference with regards to the ones of the 2 preceding years (Table 1).

The number of cancelled transplants during the COVID-19 lockdown period reached the number of 103. On the other hand, in the same timeframe of 2019, only 27 cancelled procedures were registered, and 35 in 2018. The analysis of variance revealed a significant difference (ANOVA: *p* < 0.0001). Similarly, the number of wasted corneas (not used) due to delayed or cancelled surgeries was 421, resulting in a considerable increase as compared to the previous 2 years (2019: + 528%; 2018: + 401%). The Friedman test hence resulted in a significant difference (ANOVA: *p* = 0.0035). In addition, the requested and delivered albeit not implanted corneal tissues reached a total of 9 during the lockdown period. This number resulted to be lower than the one recorded in 2019 and in 2018 (26 and 24, respectively) (*p* = 0.1024).

## Corneal transplant data during the first month after the lockdown period

The total number of donor corneas collected and harvested from May 1, 2020 to May 31, 2020 was 850. This value was notably lower than the one registered in the same period of both 2019 (− 49%) and 2018 (− 42%) (ANOVA: *p* = 0.0003).

In the aforementioned timeframe, 14 tissues (2%) were found to be retrieved from donor with proved COVID-19 or donor with unproved infection but pathological history consistent of COVID-19.

In the first month post-lockdown, 430 corneal transplants were performed, with a significant reduction as compared to the same period of 2019 (− 42%) and 2018 (− 40%) (ANOVA: *p* < 0.0001). Specifically, the most statistically significant fall was registered in the number of PKs and EKs, while no statistical differences emerged from the comparison of the total amount of ALKs, and EPs performed, as shown in Table 2 (PK reduction: − 54% when compared with 2019 and − 52% in 2018; ALK reduction: − 32% when compared with 2019 and − 32% in 2018; EK reduction: − 34% when compared with 2019 and − 31% in 2018; EP reduction: − 51% when compared with 2019 and -50% compared with 2018).

**Table 2:** Corneal transplant metrics during the first month after the lockdown period (May, 2020) vs. May, 2019 and May, 2018

	May 2020	May 2019	May 2018	*p*
N° of received corneas	850	1655	1477	0.0003
N° of Surgical Procedures	430	746	717	< 0.0001
N° of PKs	150	325	310	0.0041
N° of ALKs	54	80	80	0.2307
N° of EKs	226	341	327	0.0144
N° of EPs	17	35	34	0.6832
N° of cancelled procedures	8	7	20	0.6368
N° of requested albeit not transplanted corneas	0	7	10	0.0237
N° of wasted Grafts	65	40	58	0.9138

*PK* penetrating keratoplasty; *ALK* Anterior lamellar keratoplasty; *EK* endothelial keratoplasty

Significant difference emerged from the analysis of the amount of both the requested and delivered albeit not implanted corneal tissues (2020 = 0; 2019 = 7; 2018 = 10; ANOVA: *p* = 0.0237) while none was found regarding the proportion of cancelled procedures (2020 = 8; 2019 = 7; 2018 = 20; ANOVA: *p* = 0.6368). Overall, 65 stored corneas were lost for not being used within the expected period, a percentage marginally higher than that of the same period in 2019 and 2018 (2020 = 65; 2019 = 40; 2018 = 58; ANOVA: *p* = 0.9138).

## Lockdown period vs. first month after the lockdown period

The comparative analysis of the data related to the lockdown period as compared to the one of the first month post-lockdown revealed no statistically significant differences in terms of number of donor corneas not feasible to transplant per high-COVID-19-transmission risk and total number of ALKs, EKs and EPs, as shown in Table 3. In April, the number of surgical procedures appeared to be subjected to a significant reduction when compared to both March and May (*p* = 0.0028). Even the amount of collected corneas appeared to be lower in April than March and May (*p* = 0*.*0038). Fewer corneal grafts were lost in May than during the preceding 2 months (421 *vs*. 65; *p* = 0.0403). No differences were found in the total number of cancelled procedures (*p* = 0.0608) while a lower amount of not-transplanted corneal grafts was registered in May than during the preceding 2 months (*p* = 0.0302).

**Table 3:** Corneal transplant metrics during the lockdown period (March and April, 2020) as compared to May, 2020

	March 2020	April 2020	May 2020	*p*
N° of received corneas	789	495	850	0.0038
N° of COVID-19 positive donor corneas	21	10	14	0.6514
N° of surgical procedures	379	155	430	0.0028
N° of PKs	188	59	150	0.0337
N° of ALKs	30	19	54	0.0743
N° of EKs	161	77	226	0.2938
N° of EPs	12	18	17	0.4013
N° of cancelled procedures	93	10	8	0.0608
N° of requested albeit not transplanted corneas	5	4	0	0.0302
N° of waisted grafts	282	139	65	0.0403

*PK* penetrating keratoplasty; *ALK* Anterior lamellar keratoplasty; *EK* endothelial keratoplasty

## Discussion

This comprehensive, national cohort study provides a comprehensive and in-depth overview of the impact of COVID-19 pandemic on the Italian National Eye Bank organization and on the related surgical activity. Italy has been one of the first western countries to be affected by SARS-CoV-2 with subsequent restrictions to control its outbreak. (Romano et al. 2020; Vanni et al. 2020a).

Our survey, which included data from all the Italian Eye Bank establishments, confirms that their activities underwent profound changes throughout the country with unexpected sequelae to both the collection of eye tissues (i.e., tissues collection and harvesting), their distribution to the ophthalmology wards and their usage for surgical purpose.

It must be considered that, apart from a small percentage of cases (i.e., corneal perforation), corneal transplants are in general elective surgeries. In Italy, starting from March 9, 2020 (the starting date of the lockdown period, as for the Italian Government declaration), elective surgeries in all specialties, ophthalmology included, have been suspended in the majority of cases in order to avoid SARS-CoV-2 spreading. (Buonomo et al. 2020) A dramatic reduction in the number of keratoplasties derived, with at least 2 main related consequences.

First of all, the Eye Bank organization works as a complex structure responsible for collection, evaluation, preservation and distribution of corneal tissue. (Chaurasia et al. 2020) To ensure the proper functioning of this dynamic apparatus, a multitude of clinical and laboratory skills and economic input are required. (Bohringer et al. 2009; Shinozaki et al. 1997) Corneal tissues must be used within a maximum of 34 days from collection and the maximum storage time depends on the preservation technique used: either hypothermic storage (4–6 °C) or organ culture (31–37 °C). The latter is the most commonly used storage technique in Europe and, despite allowing a longer storage time, during the lockdown timeframe in Italy up to 421 corneas available for transplantation were discarded, having not been used before the expiry date, representing a significant waste of resources. (Jones et al. 2009; Parekh et al. 2020) However, it must be noted that, even if in a small number of cases, discarded corneas were used for research purpose, whenever authorized by local ethical committee and in accordance to the donors’ will.

Moreover, and perhaps more crucially, the social implications are profound and multifaceted, due to the recent technical innovations introduced in corneal keratoplasty and the notable impact of functional visual improvement transplants can provide in the majority of cases. (Dunker et al. 2020; Woo et al. 2019) Thus, used grafts at this time is a significant loss to the potential benefits if could have given the recipients.

Until now, the COVID-19 pandemic has harmed the overall corneal transplant activity across Italy, with a reduction of nearly 60% as compared to the same period of 2019. This obvious decrease has been mainly due to the profound reorganization of the national health service leading to the conversion of operating theatres into intensive care unit wards, permitting only urgent surgeries.

Surprisingly, a slight increase in the number of exported grafts (15%) has been reported during the analysed timeframe of 2020. This apparently counterintuitive evidence might be explained by the fact that Italy was the first European country to be subjected to COVID-19 lockdown rules, while the corneal transplant activities unrestrictedly went on in different other parts of Europe.

The current available data are representative of a whole nation Eye Bank establishment and may be considered suggestive not only of the present but especially of the forthcoming difficulties the Eye Bank facilities are going to experience. Hence, it is advisable a general re-arrangement of the Eye Bank institutions all around the world will be carried out, as the current pandemic is exposing the frailty of an already vulnerable system. In addition, since the current evolving pandemic is determining gross restrictions in corneal graft availability, ophthalmologists will need to use even more stringent prioritisation criteria to select host patients to treat in accordance to visual residue, age and functional need.
